# Reactive Oxygen Species in Host Plant Are Required for an Early Defense Response against Attack of *Stagonospora nodorum* Berk. Necrotrophic Effectors SnTox

**DOI:** 10.3390/plants10081586

**Published:** 2021-07-31

**Authors:** Svetlana Veselova, Tatyana Nuzhnaya, Guzel Burkhanova, Sergey Rumyantsev, Igor Maksimov

**Affiliations:** 1 Institute of Biochemistry and Genetics, Ufa Federal Research Centre, Russian Academy of Sciences, Prospekt Oktyabrya, 71, 450054 Ufa, Russia; tanyawww89@mail.ru (T.N.); guzel_mur@mail.ru (G.B.); rumyantsev-serg@mail.ru (S.R.); igor.mak2011@yandex.ru (I.M.); 2 Ufa Institute of Biology, Ufa Federal Research Centre, Russian Academy of Sciences, Prospekt Oktyabrya, 69, 450054 Ufa, Russia

**Keywords:** hydrogen peroxide, necrotrophic effectors-triggered susceptibility, peroxidase, redox-metabolism, septoria nodorum blotch, *Triticum aestivum*, *Tsn1*–SnToxA, *Snn3*–SnTox3 and *Snn1*–SnTox1 interactions

## Abstract

Reactive oxygen species (ROS) play a central role in plant immune responses. The most important virulence factors of the *Stagonospora nodorum* Berk. are multiple fungal necrotrophic effectors (NEs) (SnTox) that affect the redox-status and cause necrosis and/or chlorosis in wheat lines possessing dominant susceptibility genes (*Snn*). However, the effect of NEs on ROS generation at the early stages of infection has not been studied. We studied the early stage of infection of various wheat genotypes with *S nodorum* isolates -Sn4VD, SnB, and Sn9MN, carrying a different set of NE genes. Our results indicate that all three NEs of SnToxA, SnTox1, SnTox3 significantly contributed to cause disease, and the virulence of the isolates depended on their differential expression in plants (*Triticum aestivum* L.). The *Tsn1*–SnToxA, *Snn1*–SnTox1and *Snn3*–SnTox3 interactions played an important role in inhibition ROS production at the initial stage of infection. The *Snn3*–SnTox3 inhibited ROS production in wheat by affecting NADPH-oxidases, peroxidases, superoxide dismutase and catalase. The *Tsn1*–SnToxA inhibited ROS production in wheat by affecting peroxidases and catalase. The *Snn1*–SnTox1 inhibited the production of ROS in wheat by mainly affecting a peroxidase. Collectively, these results show that the inverse gene-for gene interactions between effector of pathogen and product of host sensitivity gene suppress the host’s own PAMP-triggered immunity pathway, resulting in NE-triggered susceptibility (NETS). These results are fundamentally changing our understanding of the development of this economical important wheat disease.

## 1. Introduction

Plants have developed several levels of defense against microbial pathogens, which have been described in the “zig-zag” model of the plant immune system [1]. The first line of defense in plants is through the perception of pathogen-associated molecular patterns (PAMPs) by pattern recognition receptors (PRRs), which leads to development of basal immunity, known as PAMP-triggered immunity (PTI) [1]. However, the pathogen can suppress PTI using effectors which leads to the development of effector-triggered susceptibility (ETS). The second line of defense in plants is called effector-triggered immunity (ETI) and develops when an effector is recognized by products of effector-specific resistance genes, the most common being the so-called nucleotide-binding and leucine-rich repeat domain proteins (NB-LRR class) [1]. The development of PTI and ETI induces similar responses in plants: both lines of defense can be separated in time and space, but both are closely related to the production of reactive oxygen species (ROS) [2]. PTI develops in the first minutes and hours of infection and is a non-specific reaction. PTI is the result of activating a plant response involving an oxidative burst, induction of mitogen-activated protein kinase (MAPK) cascade pathways and transcriptional activation of defense response genes [2]. PAMP-triggered ROS perform two functions: (1) the apoplastic ROS are cytotoxic and kill pathogens, (2) ROS act as signaling molecules to activate plant defenses [2]. ETI develops later and is a specific gene-for-gene response. The development of ETI leads to oxidative burst and formation of necrosis restricting growth of the biotrophic pathogens [1].

Until recently, necrotrophic pathogens were considered as universal and non-host specific. However, recent studies have revealed that about 20 necrotrophic fungal species in the Dothideomycete class produced effector proteins also known as host-selective toxins (HSTs) or necrotrophic effectors (NEs) that interact either directly or indirectly with dominant sensitivity/susceptibility gene products to induce disease [3]. When a specific NE is recognized by the corresponding host gene, a host response follows that allows necrotrophs to penetrate, grow and sporulate. The lack of NE recognition by the host leads to resistance. Therefore, these host–pathogen interactions are controlled in an inverse gene-for-gene manner, and the dominant alleles of the host NEs recognition genes are considered as susceptibility genes [3]. Molecular cloning of several plant NEs’ susceptibility genes disclosed that they have NB and LRR domains and are the classic resistance genes involved in ETI [4,5]. Additional studies of host response to recognition of NE discovered hallmarks of an ETI response [5]. Thus, NEs use the host’s ETI pathway to develop sensitivity, resulting in NE-triggered susceptibility (NETS) [3,5]. Classical HST pathogens include species of *Cochliobolus*, *Alternaria*, *Pyrenophora* and *Stagonospora* [3].

Pathogenic fungus *Stagonospora nodorum* Berk. (syn. *Septoria*, *Parastagonospora*; *teleo*, *Phaeosphaeria*) is the causative agent of Septoria nodorum blotch (SNB) of wheat. The most important factors of *Stagonospora nodorum* virulence include numerous fungal NEs encoded by *SnTox* genes [6,7]. To date, three effector genes have been identified in the genome of *S. nodorum* (*SnToxA*, *SnTox1*, *SnTox3*) [6]. *SnToxA* encodes a 13.2 kDa mature protein that causes chlorosis in wheat varieties that contain *Tsn1* located on wheat chromosome 5BL [4]. *SnTox1* encodes a 10.3 kDa mature cysteine-rich protein with a chitin-like binding motif at the C-terminus. Sensitivity to SnTox1 is defined by the *Snn1* gene located on wheat chromosome 1BS [6,8]. Both *Tsn1* and *Snn1* have been cloned and encode a serine/threonine protein kinase, nucleotide binding site leucine rich repeat protein (NB-LRR) and a wall-associated kinase protein (WAK), respectively [5,7]. *SnTox3* is an intron-free gene that codes for a 17.5 kDa mature protein with six cysteine residues. Three disulfide bridges formed by six cysteine residues in SnTox3 are essential for structure and function of the effector protein [6,9]. Sensitivity to SnTox3 is conferred by *Snn3-B1* and *Snn3-D1* located on wheat chromosomes 5BS and 5DS, respectively [6].

Effectors SnToxA, SnTox1, SnTox3 are considered the main ones in the pathogen *S. nodorum* and are quite widespread among strains and isolates [8]. Effectors SnToxA, SnTox1, SnTox3 cause necrosis and chlorosis in susceptible wheat genotypes, it follows from this that they have an impact on the redox metabolism of the host plant. Unfortunately, the whole signal transduction pathway from recognition of the effector by the receptor to the necrosis development is unknown [5,7]. However, the role of each compatible interaction, *Tsn1*–SnToxA, *Snn3*–SnTox3, and *Snn1*–SnTox1, in the suppression of PTI and the development of ETS is expected [7]. First of all, PTI is characterized by rapid and strong production of ROS (mainly hydrogen peroxide (H_2_O_2_) and superoxide radical) in the apoplast through activation of NADPH-oxidases localized on the plasmallema also known as respiratory burst oxidase homologs (RBOHs), as well as peroxidases (POD) and superoxide dismutase (SOD), leading to PTI-dependent basal defenses that inhibit invasion of pathogens [2,10,11,12]. It is believed that suppression of primary ROS burst during PTI by effector proteins as virulence factors is a common adaptation of many virulent pathogens [2]. Much data have been accumulated on the effectors of various pathogens that suppress ROS burst during PTI [2]. However, there is no such data on NEs SnToxA, SnTox1, and SnTox3.

In this study, our aim was to study the effects of *S. nodorum* effectors SnToxA, SnTox1, SnTox3 on the development of disease symptoms, generation of hydrogen peroxide, expression of oxidoreductase genes and the activity of their protein products at an early stage of infection in various cultivars Zhnitsa, Kazakhstanskaya 10, Omskaya 35 of bread spring wheat (*Triticum aestivum* L.) infected with isolates of *S. nodorum*—Sn4VD, SnB and Sn9MN—carrying a various set of NE genes. Here, we evaluated the roles of each compatible interaction *Tsn1*–SnToxA, *Snn3*–SnTox3, *Snn1*–SnTox1 in suppressing ROS production at the initial stage of infection.

## 2. Results

### 2.1. Analysis of the Necrotrophic Effector (NE) Sensitivity Genes Tsn1, Snn3-B1, and Snn1 in Three Cultivars of Bread Spring Wheat

**Figure 1:**
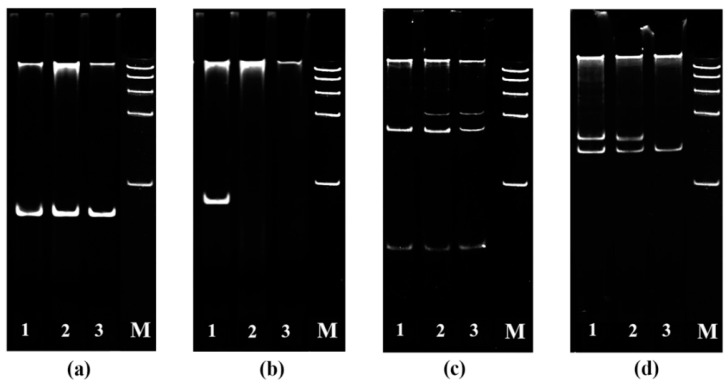
Identification of alleles of susceptibility genes *Snn1* (**a**), *Tsn1* (**b**) with using primers for the intragenic markers and *Snn3-B1* locus *Table* (**c**,**d**) using primers for SSR markers Xcfd20 (**c**) and Xgwm234 (**d**) in different wheat cultivars by PCR. 1—Zhnitsa; 2—Kazakhstanskaya 10; 3—Omskaya 35; M—DNA molecular weight ladder 100–1000 bp.

The *Tsn1*–SnToxA, *Snn3*–SnTox3 and *Snn1*–SnTox1 interactions were investigated in this study. Alleles of susceptibility genes *Snn1*, *Tsn1* and locus *Snn3-B1* were identified by PCR in three cultivars of bread spring wheat (*Triticum aestivum* L.) Zhnitsa (Zh), Kazakhstanskaya 10 (Kaz10), Omskaya 35 (Om35) (Figure 1).

The dominant allele of the *Snn1* gene was found in all three studied varieties (Figure 1a). The dominant allele of the *Tsn1* gene was found in one variety—Zhnitsa (Figure 1b). The null-allele of the *Snn3-B1* locus was not found in these varieties. However, the varieties differed in the allelic composition of the *Snn3-B1* locus (Figure 1c,d). The *Xgwm234* marker was represented by one allele in the resistant variety Om35, and it was represented by two alleles in the susceptible cultivars Kaz10 and Zhnitsa (Figure 1d), which may indicate the insensitivity or sensitivity of the varieties to NE SnTox3 [13]. Thus, Om 35 (*tsn1/Snn1/snn3*) was sensitive to NE of SnTox1. Variety Kaz10 (*tsn1/Snn1/Snn3*) was sensitive to NE of SnTox1 and SnTox3. The Zhnitsa (*Tsn1/Snn1/Snn3*) was sensitive to all three effectors.

### 2.2. Analysis of Necrotrophic Effectors Genes of SnTox1, SnToxA, and SnTox3 in S. nodorum Isolates Sn4VD, SnB and Sn9MN

**Figure 2:**
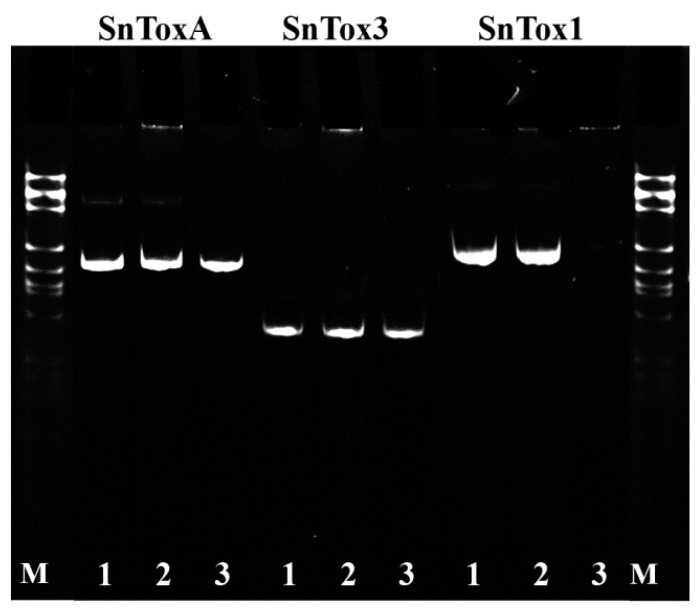
Identification of the *SnToxA*, *SnTox3* and *SnTox1* genes by PCR in tree isolates of *S. nodorum*: 1. Sn4VD; 2. Sn9MN; 3. SnB; M—DNA molecular weight ladder 100–1000 bp.

Three *S. nodorum* isolates Sn4VD, SnB and Sn9MN were tested for presence of the three NE genes *SnToxA*, *SnTox3* and *SnTox1* by PCR (Figure 2). The isolates Sn4VD and Sn9MN contained three NE genes *SnToxA, SnTox3* and *SnTox1* in their genome (Figure 2). The isolate SnB contained only two NE genes *SnToxA* and *SnTox3* in the genome (Figure 2).

Investigation of the transcriptional activity of NE genes in three *S. nodorum* isolates SnB, Sn4VD, and Sn9MN during inoculation of three wheat genotypes Zhnitsa, Kaz10 and Om35 revealed differences in expression (Table 1). Previously, it was shown that the pathogen actively began to penetrate into plant tissues and induce a PTI response after 6 h of infection; after 24 h of infection, the pathogen induced an ETS and ETI response in susceptible and resistant genotypes, respectively [14]. After 72–96 h of infection, we observed the appearance of small necrosis on the leaves [14]. Therefore, we investigated the expression of NE genes at 6, 24 and 72 h after infection (Table 1).

When wheat plants were inoculated with the Sn4VD isolate, the mRNA abundance of all three NE genes was not detected in three cultivars (Table 1). The *SnTox1* gene has not been detected in the genome of the SnB isolate (Figure 2). Therefore, upon inoculation with isolate SnB of all three cultivars, the content of mRNA of the *SnTox1* gene was not found (Table 1). The transcript level of *SnToxA* gene was significantly higher in both SnB and Sn9MH isolates upon inoculation of SnToxA-sensitive cv. Zhnitsa (*Tsn1/Snn1/Snn3*) than in the case of inoculation of SnToxA-insensitive Kaz10 and Om35 cultivars lacking the corresponding susceptibility gene (Table 1).

Expression of the *SnTox1* gene in planta was detected only in the Sn9MN isolate (Table 1). All three cultivars Zhnitsa, Kaz10, and Om35 were SnTox1-sensitive; however, the accumulation of mRNA of this gene in cultivars was different (Table 1). The maximum mRNA abundance of the *SnTox1* gene was found during inoculation of Kaz10 and Zhnitsa, and the minimum mRNA abundance was observed during inoculation of cv. Om35 (Table 1). The transcript level of *SnTox3* gene upon inoculation of the SnTox3-insensitive cv. Om35 was lower than in SnTox3-sensitive Zhnitsa and Kaz10 cultivars in both isolates SnB and Sn9MN (Table 1). Interestingly, the mRNA abundance of *SnTox3* gene was high in isolate SnB upon inoculation of both SnTox3-sensitive cultivars Zhnitsa and Kaz10, but the expression of *SnTox3* gene in isolate Sn9MN in planta was suppressed upon inoculation of cv. Kaz10 and was activated upon inoculation of cv. Zhnitsa (Table 1). Our results showed that the expression of *SnToxA* and *SnTox1* genes was significantly activated after 6 h of infection in sensitive genotypes, while the expression of the *SnTox3* gene reached its maximum only by 72 h of infection (Table 1).

### 2.3. The Role of Compatible Interactions Tsn1–SnToxA, Snn3–SnTox3, Snn1–SnTox1 in Causing Disease

**Figure 3:**
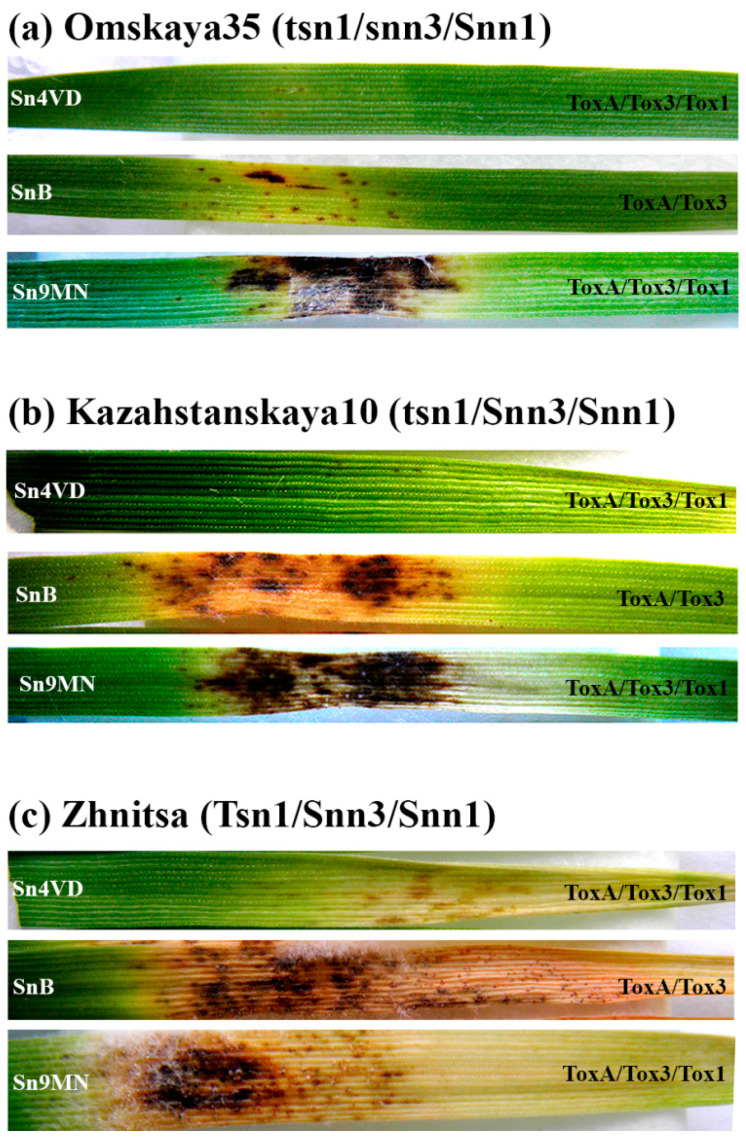
Analysis of phenotypes of gene-for-gene interactions in various compatible and incompatible combinations in the *S. nodorum*–*T. aestivum* pathosystem. Photographs represent results of a typical variant from a series of experiments. Experiments were carried out on the separated first leaves. The development of symptoms was recorded 6 days after infection of three bread spring wheat varieties with different allelic states of susceptibility genes Omskaya 35 (*tsn1/snn3/Snn1*) (**a**), Kazakhstanskaya 10 (*tsn1/Snn3/Snn1*) (**b**) and Zhnitsa (*Tsn1/Snn3/Snn1*) (**c**) with three isolates of *S. nodorum* SnB, Sn9MN, Sn4VD carrying a different set of NE genes *SnToxA*, *SnTox3* and *SnTox1*.

Thus, incompatible interactions or resistance were observed when cultivars were inoculated with the Sn4VD isolate, because the isolate did not produce NEs (Table 1). The incompatible interaction was observed in the combination cultivar/isolate Om35/SnB (*tsn1/snn3/Snn1*–*ToxA/Tox3/tox1*). The minimal damage areas were observed in all incompatible interactions in variants of Om35/Sn4VD, Kaz10/Sn4VD, Zh/Sn4VD, Om35/SnB (Table 2, Figure 3).

The *Snn1*–SnTox1 interaction and the effect of SnTox1 on the development of the disease were observed in the combination cultivar/isolate Om35/Sn9MN (the combination of genotypes is presented in Table 2). The *Snn1*–SnTox1 interaction led to the formation of necrosis, occupying about 25% of the leaf area and did not cause the formation of chlorosis (Table 2, Figure 3). The *Snn3*–SnTox3 interaction and the effect of SnTox3 on disease progression were observed in combination cultivar/isolate Kaz10/SnB (Table 2). The *Snn3*–SnTox3 interaction led to the formation of necrosis and significant chlorosis, together occupying more than 50% of the leaf area (Table 2, Figure 3).

The *Snn3*–SnTox3 and *Snn1*–SnTox1 interactions were observed in combination cultivar/isolate Kaz10/Sn9MN (Table 2). However, we did not find the expected significant increase in the total lesion, but there was an increase in necrotic spots compared to the Kaz10/SnB variant (*Snn3*–SnTox3) (Table 2, Figure 3). The *Tsn1*–SnToxA and *Snn3*–SnTox3 interactions were observed in combination cultivar/isolate Zh/SnB (Table 2). These interactions led to a significant increase in the damage areas compared to the Kaz10/SnB variant (*Snn3*–SnTox3), chlorosis and necrosis occupied more than 70% of the leaf area (Table 2, Figure 3). The *Tsn1*–SnToxA, *Snn3*–SnTox3, *Snn1*–SnTox1 interactions were observed in combination cultivar/isolate Zh/Sn9MN (Table 2). In this variant, an increase in the affected areas was found in comparison with the Zh/SnB variant (*Snn3*–SnTox3 and *Tsn1*–SnToxA), mainly due to an increase in the formation of necrosis (Table 2, Figure 3). Thus, according to our results, NE SnTox1 caused the development of necrosis, NE SnTox3 stimulated the development of both necrosis and chlorosis, and NE SnToxA induced the development of extensive chlorosis in susceptible wheat plants.

### 2.4. The Role of Compatible Interactions in Suppression of Reactive Oxygen Species (ROS) Production

To check the role of compatible interactions in suppression of ROS production we studied two isoforms of wheat NADPH oxidase, RBOHD and RBOHF, which are not comprehensively studied, akin to the *AtRboh* Arabidopsis genes. Nevertheless, there are works showing that the *TaRbohD* and *TaRbohF* genes, which are homologues of the Arabidopsis genes *AtRbohD* and *AtRbohF* and are activated under biotic stress, are involved in the production of ROS [15]. In addition, we investigated the expression of genes encoding superoxide dismutase (*TaSod*) and anionic peroxidase (*TaPrx*). We also studied the activity of peroxidases and catalases (CATs) in all incompatible and compatible interactions.

**Figure 4:**
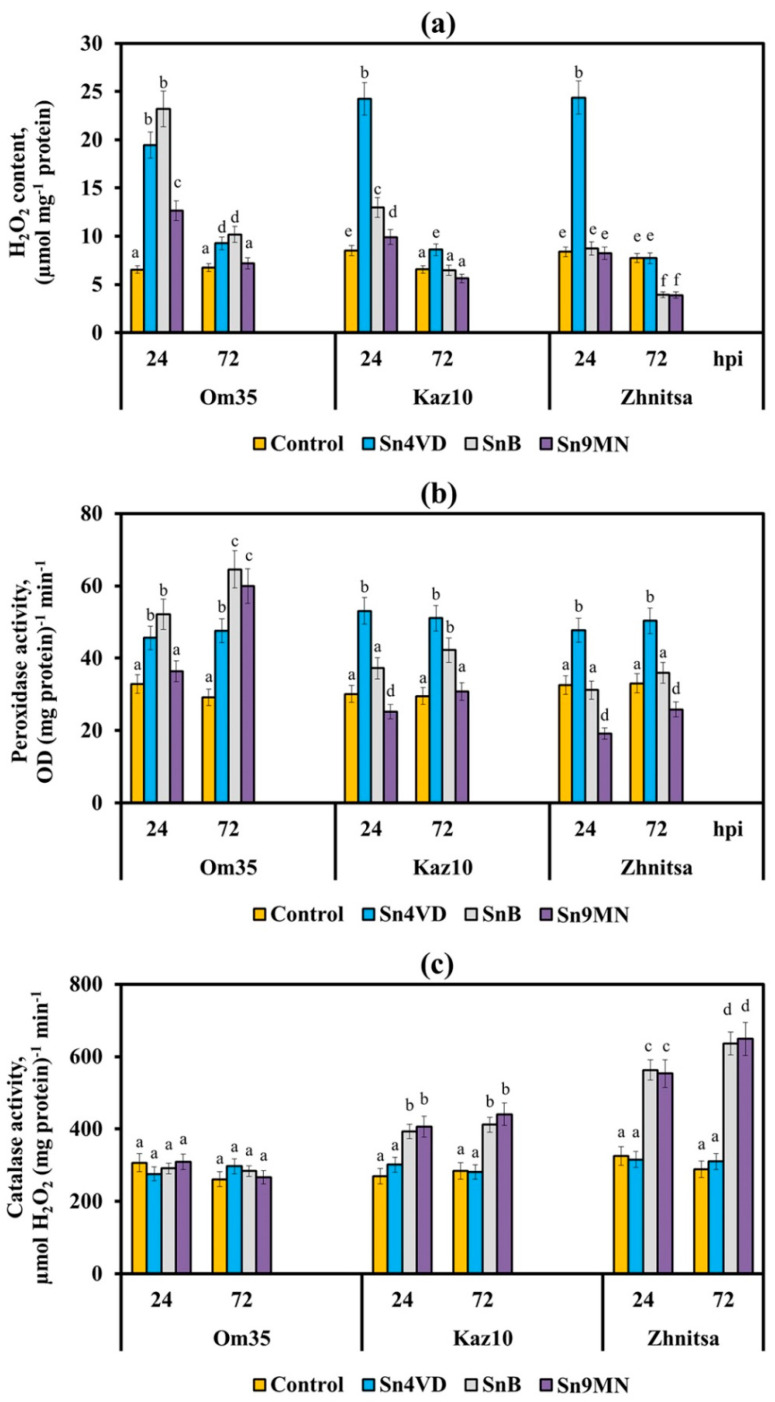
Hydrogen peroxide contents (**a**), enzymes activities of peroxidase (**b**) and catalase (**c**) in leaves of wheat cultivars Om35, Kaz10 and Zhnitsa 24 and 72 h post-inoculation (hpi) with *S. nodorum* isolates Sn4VD, SnB and Sn9MN. The samples are indicated as follows: Control—non-infected plants; Sn4VD—plants infected with isolate Sn4VD; SnB—plants infected with isolate SnB; Sn9MN—plants infected with isolate Sn9MN. Means ± SE (n = 6) marked with similar Latin letters do not differ significantly according to the LSD-test (*p* ≤ 0.05).

**Figure 5:**
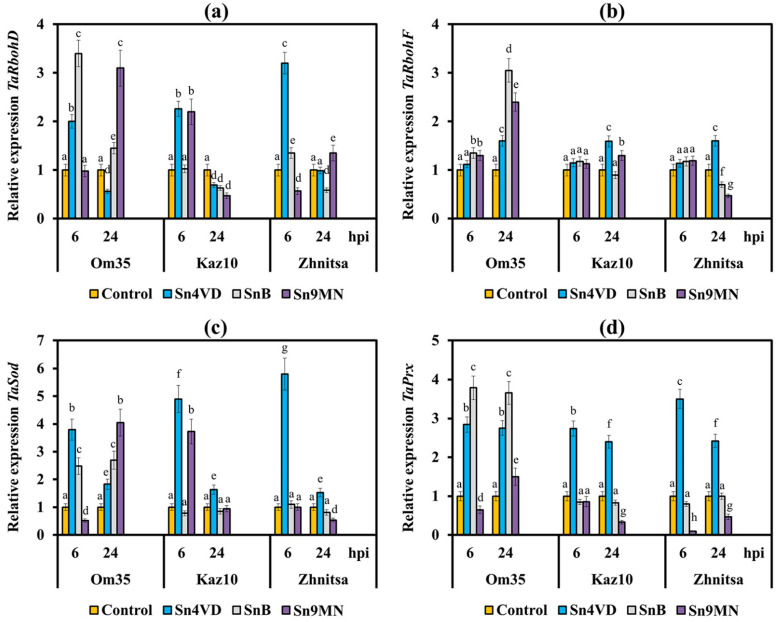
The relative expression of genes encoding isoforms of wheat NADPH oxidase, RBOHD and RBOHF, as well as superoxide dismutase and anionic peroxidase in three wheat cultivars Om35, Kaz10 and Zhnitsa 6 and 24 h post-inoculation (hpi) with *S. nodorum* isolates Sn4VD, SnB and Sn9MN. (**a**) The mRNA level of the *TaRbohD* gene. (**b**) *TaRbohF* gene. (**c**) *TaSod* gene. (**d**) *TaPrx* gene. Expression values were normalized to the housekeeping gene *TaRLI* as an internal reference and presented relative to the normalized expression levels in mock-treated control plants (Control). The samples are indicated as follows: Control—non-infected plants; Sn4VD—plants infected with isolate Sn4VD; SnB—plants infected with isolate SnB; Sn9MN—plants infected with isolate Sn9MN. Means ± SE (n = 6) marked with similar Latin letters do not differ significantly according to the LSD-test (*p* ≤ 0.05).

Earlier, we found that the resistance of the *T. aestivum* to the pathogen *S. nodorum* was determined by the intensive generation of ROS, mainly H_2_O_2_, due to an increase in POD activity and a decrease or absence of an increase in CAT activity in the initial stage of infection [14,16]. In this study, the threefold and fourfold increase in the H_2_O_2_ content was observed in variants with incompatible interactions (Om35/Sn4VD, Kaz10/Sn4VD, Zh/Sn4VD, Om35/SnB) (Figure 4a) at an early stage of infection. The oxidative burst in all incompatible interactions was accompanied by a significant increase in the activity of free PODs, inhibition of CAT activity (Figure 4b,c), and an increase in the transcripts level of genes *TaRbohD, TaRbohF, TaSod* and *TaPrx* (Figure 5). However, the transcript level of the *TaRbohF* gene was much higher in the Om35/SnB variant upon inoculation with a virulent isolate SnB than upon inoculation of plants with an avirulent isolate Sn4VD (Om35/Sn4VD, Kaz10/Sn4VD, Zh/Sn4VD) (Figure 5b). Thus, a typical reaction of resistance to *S. nodorum* was observed in all incompatible interactions, which led to the development of an oxidative burst and restriction of the pathogen growth.

All compatible interactions inhibited H_2_O_2_ production in susceptible wheat varieties at an early stage of infection compared with incompatible interactions (Figure 4a). The *Snn1*–SnTox1 (Om35/Sn9MN) and *Snn3*–SnTox3 (Kaz10/SnB) interactions reduced H_2_O_2_ production by two times as compared to the incompatible interaction in variant Om35/SnB (Figure 4a). The presence of two or three compatible interactions in wheat plants leads to an even greater decrease in H_2_O_2_ production (Figure 4a).

The *Snn3*–SnTox3 interaction (Kaz10/SnB) inhibited the H_2_O_2_ production due to a decrease in the transcripts level of genes *TaRbohD, TaRbohF, TaSod* and *TaPrx* and POD activity, as well as due to an increase in CAT activity (Figure 4 and Figure 5). It should be noted that the *Snn1*–SnTox1 compatible interaction (Om35/Sn9MN) did not lead to an increase in CAT activity, a decrease in the transcript level of *TaRbohF* gene and a reduction in the mRNA content of *TaRbohD* and *TaSod* genes 24 h after infection in comparison with another compatible interaction *Snn3*–SnTox3 (Kaz10/SnB) (Figure 4 and Figure 5). The transcript levels of the gene *TaRbohF* and CAT activity in variants Om35/SnB and Om35/Sn9MN were also found to be similar (Figure 5b).

Our results also showed that CAT activity and *TaRbohF* gene expression in the Kaz10/Sn9MN variant (*Snn3*–SnTox3, *Snn1*–SnTox1) were similar to the Kaz10/SnB variant (*Snn3*–SnTox3) (Figure 4c and Figure 5b). At the same time, in the Kaz10/Sn9MN variant, the transcripts level of *TaRbohD* and *TaSod* genes increased and was comparable with incompatible interactions (Om35/Sn4VD, Kaz10/Sn4VD, Om35/SnB); by contrast, the POD activity and the accumulation of mRNA of the *TaPrx* gene were inhibited much more strongly than in the Kaz10/SnB variant (Figure 5).

Therefore, the *Snn1*–SnTox1 interaction did not affect the isoforms of wheat NADPH oxidase, RBOHD and RBOHF, as well as SOD and CAT, but significantly inhibited the expression of the *TaPrx* gene encoding the anionic peroxidase and decreased the activity of the POD enzyme itself (Figure 4 and Figure 5). It should be noted that in the Om35/Sn9MN variant, the *Snn1*–SnTox1 interaction did not inhibit POD activity as compared to the Kaz10/Sn9MN interaction, which could be associated with the low expression of the *SnTox1* gene in this variant (Om35/Sn9MN) (Figure 4b, Table 1).

The Zh/SnB variant (*Snn3*–SnTox3, *Tsn1*–SnToxA) slightly differed from the Kaz10/SnB variant (*Snn3*–SnTox3) in its effect on transcripts level of *TaRbohD*, *TaRbohF*, *TaSod*, and *TaPrx* genes (Figure 5); however, it more strongly inhibited POD activity and much more strongly increased CAT activity (Figure 4). These results indicate the predominant effect of the *Tsn1*–SnToxA interaction on the activity of POD and CAT. The Zh/Sn9MN variant (*Snn3*–SnTox3, *Tsn1*–SnToxA, *Snn1*–SnTox1) more strongly inhibited POD activity and reduced the mRNA content of *TaPrx*, *TaRbohD*, *TaRbohF*, and *TaSod* genes as compared to the Zh/SnB variant (*Snn3*–SnTox3, *Tsn1*–SnToxA) (Figure 4 and Figure 5). These results suggest an additive effect of the triple compatible interaction (*Snn3*–SnTox3, *Tsn1*–SnToxA and *Snn1*–SnTox1) in the Zh/Sn9MN variant on the inhibition of H_2_O_2_ production.

## 3. Discussion

### 3.1. Expression of Necrotrophic Effectors Determines the Virulence of the S. nodorum Isolate

The interaction in the wheat–*S. nodorum* pathosystem is of the gene-for-gene type [7]. To date, eight *Snn*–SnTox interactions are known [17]. In addition to the three main interactions *Snn1*–SnTox1, *Snn3*–SnTox3, and *Tsn1*–SnToxA, several other interactions have been identified, such as *Snn2*–SnTox2, *Snn4*–SnTox4, *Snn5*–SnTox5, *Snn6*–SnTox6 and *Snn7*–SnTox7 [18,19,20,21,22,23]. The effect of each *Snn*-SnTox interaction is incomplete and is complemented by other interactions. However, the overall host response to *S. nodorum* lesions does not always strictly follow the inverse gene-for-gene model, and sometimes the severity of the disease is not a function of the number of *Snn*–SnTox interactions. Thus, 10% of interactions with atypical symptoms are encountered when analyzing large collections, [24]. The mechanisms of all discovered variants of *Snn*–SnTox relationships available in the literature are not fully disclosed, but it is assumed that multiple *Snn*-SnTox interactions can exhibit both additivity and epistasis [6]. In addition, a variety of manifestations of one relationship can be caused by different expression level of NE genes upon infection of a plant sample [6]. Thus, analysis of NE gene-knockout isolates showed that the effect of some interactions could be masked or inhibited by other compatible interactions, and the regulation of this occurs at the level of NE gene transcription [17].

In our work, we used different wheat genotypes Omskaya 35 (*tsn1/snn3/Snn1*), Kazahstanskaya 10 (*tsn1/Snn3/Snn1*), Zhnitsa (*Tsn1/Snn3/Snn1*) and three *S. nodorum* isolates Sn4VD (*toxa/tox3/tox1*), SnB (*ToxA/Tox3/tox1*), Sn9MN (*ToxA/Tox3/Tox1*), carrying a different set of susceptibility genes and NE genes, respectively to study three interactions *Snn1*–SnTox1, *Snn3*–SnTox3, and *Tsn1*–SnToxA,. Analysis of the transcriptional activity of the NE genes (Table 1) and the assessment of the damage areas (Table 2) revealed a relationship between the virulence of the isolate and the expression of the NE genes, and also revealed epistatic and additive interactions. Thus, we assume that the avirulence of isolate Sn4VD, manifested in the absence of visible lesions on the leaves of all studied cultivars was associated with the absence of expression of three NE genes upon inoculation of three different wheat genotypes (Table 1 and Table 2, Figure 3).

We also found that the intensity of *SnToxA* gene expression in two isolates, SnB and Sn9MN, depended on the presence of the possibility of a compatible interaction, which correspond with the literature data. Therefore, for the isolate Sn5, a two-fold increase in the expression of the *SnToxA* gene was shown earlier with a compatible interaction compared with an incompatible interaction [25]. In addition, the transcript level of the *SnToxA* gene in the isolate Sn9MN was 1.8 times higher than in the isolate SnB 72 h after inoculation of the susceptible cultivar Zhnitsa (Table 1). This may explain the higher aggressiveness of the isolate Sn9MN as compared to the isolate SnB (Table 2). This conclusion is consistent with the observation by some authors of a positive correlation between the expression level of *SnToxA* gene and the contribution of the *Tsn1*–SnToxA interaction to disease [25,26].

We found the same pattern of expression activation of NE genes in the case of a compatible interaction for two other effectors SnTox1 and SnTox3. In addition, the rate and degree of expression activation of the *SnTox1* gene depended on the number of compatible interactions upon inoculation of different genotypes. Thus the Om35/Sn9MN variant had one compatible interaction *Snn1*–SnTox1 and showed the lowest accumulation of *SnTox1* gene transcripts and the lowest degree of plant damage (Table 1 and Table 2, Figure 3). The Kaz10/Sn9MH variant, which had two compatible interactions *Snn1*–SnTox1 and *Snn3*–SnTox3, began to accumulate mRNA of the *SnTox1* gene only 24 h after infection (Table 1). In the Zh/Sn9MN variant with three compatible interactions *Tsn1*–SnToxA, *Snn1*–SnTox1, and *Snn3*–SnTox3, the transcripts of *SnTox1* gene accumulated after 6 h of infection, and a high level of this gene transcript was detected 72 h after infection (Table 1). This variant had the largest affected areas (Table 2, Figure 3). RNA sequencing analysis in planta showed that *SnTox1* was differentially expressed between *S. nodorum* isolates Sn4, Sn5, SN15 after infection [17]. Such a dependence of the expression of the NE gene on the number of compatible interactions may indicate the presence of a common mechanism for regulating the transcription of the NE genes. This regulatory mechanism may be associated with the activity of transcription factors that positively and negatively regulate the expression of NE genes [24,27].

The accumulation of *SnTox3* gene transcripts in compatible interactions Zh/SnB, Zh/Sn9MN, Kaz10/SnB (*Snn3*–SnTox3) was 5.5 times or more higher than in incompatible interactions Om35/SnB and Ohm35/Sn9MN (*snn3*–SnTox3) (Table 1). For the first time, we showed for two *S. nodorum* isolates SnB and Sn9MN that the wheat genotype influenced on the transcriptional activity of the *SnTox3* gene. Previously a similar effect of the wheat genotype on the transcriptional activity of the *SnToxA* gene for other *S. nodorum* isolates was found by other authors [25,26]. By contrast, we found inhibition of the transcript accumulation of the *SnTox3* gene, with a compatible interaction of Kaz10/Sn9MN (*Snn3*–SnTox3, *Snn1*–SnTox1), compared with other compatible interactions (Table 1). Perhaps this effect was a manifestation of the epistatic interaction of *Snn1*–SnTox1 in relation to *Snn3*–SnTox3, which coincides with the recently obtained results of other authors [6]. Recently, it was found that the *Snn1*–SnTox1 interaction is epistatic in relation to *Snn3*–SnTox3, and epistasis manifested itself in the differential expression of the *SnTox3* gene [6].

In addition, in the Zh/Sn9MN variant (*Snn3*–SnTox3, *Tsn1*–SnToxA and *Snn1*–SnTox1), where we found an additive effect of three compatible interactions, we did not find suppression of the transcriptional activity of the *SnTox3* gene or another effectors genes, on the contrary, the transcripts abundance of genes effectors in this variant was the largest (Table 1). The additive effect of the triple compatible interaction is shown by us for the first time. Earlier, other authors showed an additive effect for compatible interactions *Tsn1*–SnToxA and *Snn1*–SnTox1 [28].

Thus, we found that all three NEs SnToxA, SnTox3, and SnTox1 played an important role in the development of the disease in compatible interactions. Effectors SnTox3 and SnTox1 exhibited epistatic interaction, which was removed by a triple compatible interaction (*Snn3*–SnTox3, *Tsn1*–SnToxA and *Snn1*–SnTox1). We assume that, the mechanism of epistasis and additive interaction was associated with the regulation of the transcriptional activity of the NE genes; therefore, the virulence of the isolate was also associated with the transcriptional activity of the NE genes. The avirulent isolate Sn4VD had no transcripts of all three NE genes, while the virulent isolate Sn9MN had a high level of mRNA abundance of all three NE genes upon inoculation of a susceptible cultivar.

### 3.2. The S. nodorum NEs SnToxA, SnTox1 and SnTox3 Suppress the Early Defense Response of Plants Due to the Effect on the Enzymes of Redox Metabolism

Pathogens have developed numerous efficient strategies to overcome plant immunity for the establishment of compatible interactions that target the intermediates of common defense signaling pathways. The most frequent target of pathogen effectors is a powerful ROS generation system [2]. ROS produced by a plant when attacked by pathogens is a powerful weapon against pathogens [2,12]. However, pathogens effectors are able to reduce plant ROS bursts in different ways during infection. The effectors of pathogens act both in the apoplast and in the cytoplasm. Apoplastic effectors interfere with the perception of PRRs by PAMPs, preventing activation of membrane-bound NADPH oxidase RBOHs. Removal of ROS from the apoplastic area reduces its direct toxicity to pathogens, which may be beneficial for the successful colonization of pathogens. Cytoplasmic effectors target plant MAPK cascades, and WRKY transcription factors (transcription factors of WRKY family that contain WRKY domains at the N-terminus, having a conserved heptapeptide sequence WRKYGQK, and a zinc-finger-like motif at the C-terminus), suppressing the expression of RBOHs that are essential for robust ROS generation [2,29].

Our results showed that the suppression of the host defense response by the effectors of *S. nodorum* SnToxA, SnTox1, SnTox3 was carried out due to a decrease in the generation of H_2_O_2_ in the leaves of susceptible varieties at the initial stage of infection. The *Snn3*–SnTox3 interaction inhibited H_2_O_2_ production in wheat at the early stage of infection by affecting four enzymes of redox metabolism: NADPH-oxidases, peroxidases, superoxide dismutase and catalase. The *Tsn1*–SnToxA interaction suppressed the production of H_2_O_2_ by activating mainly the CAT activity and inhibiting the POD activity. It is known that CAT activate the decomposition reaction of H_2_O_2_ molecules [10] The *Snn1*–SnTox1 interaction inhibited the production of H_2_O_2_ in wheat by mainly reducing the POD activity and the transcript level of gene encoding anionic peroxidase (*TaPrx*), which is a lignin-forming peroxidase [30].

Importantly, we found that relationships between different combinations of *Snn3*–SnTox3, *Tsn1*-SnToxA and *Snn1*–SnTox1 interactions ranged from additive to epistatic. This was reflected in the effect of compatible interactions on the enzymes of redox metabolism. Thus, the absence of the effect of *Snn3*–SnTox3 interaction on the expression of *TaRbohD*, *TaRbohF* and *TaSod* genes in the Kaz10/Sn9MN variant with two interactions (*Snn3*–SnTox3, *Snn1*–SnTox1) was possibly associated with epistasis of *Snn1*–SnTox1 in relation to *Snn3*–SnTox3. On the contrary, we associate a stronger effect on all the studied components of redox metabolism in the Zh/Sn9MN variant (*Snn3*–SnTox3, *Tsn1*–SnToxA, and *Snn1*–SnTox1) with the development of the additive effect of three NE genes.

We assume that a different effect of NEs on the enzymes of redox metabolism could be associated with additional functions of effectors described recently [7]. It was discovered that SnToxA and SnTox3 directly interact with the pathogenesis-related protein 1 (PR-1), which can lead to increased susceptibility to *S. nodorum* [31]. In addition, in the case of SnTox3 with the PR-1 protein interaction, enhanced disease reaction was observed in a Snn3-dependent manner [31]. PR1 protein is a marker protein of salicylate-dependent defense response of plants against pathogens [32]. It is known that the salicylate-dependent response is associated with an oxidative burst and, first of all, with the activation of the NADPH-oxidase and apoplastic peroxidases and with inhibition of catalase activity [10].

Our results showed that the *Tsn1*–SnToxA and *Snn3*–SnTox3 interactions led to a decrease in the transcript level of genes *TaRbohD*, *TaRbohF* and *TaPrx* and an increase in the activity of CAT. This could lead to disruption and inhibition of salicylate-dependent plant defense response and an increase in the disease reaction. We have shown earlier that suppression of defense response and development of large lesions in susceptible wheat genotypes was associated with inhibition of marker genes transcription of the salicylate-signaling pathway *PR-1* and *PR-2* at the initial stage of infection [16]. In addition, we also showed, using various *S. nodorum* isolates, that NE SnTox3 decreased the transcript level of the *PR-1* and *PR-2* genes, and also inhibited transcription of the *TaWRKY13* gene markers of the salicylate signaling pathway [14]. We have proved that the salicylate signaling pathway plays a major role in the induction of defense reactions in wheat plants against the *S. nodorum* at an early stage of infection [14].

Peroxidase and catalase are important components of the salicylate signaling pathway and determine the redox status of an infected plant [12]. Effectors can affect these enzymes both directly and indirectly. It was shown using cytological and functional assays that the *Ustilago maydis* effector Pep1 (Protein essential during penetration-1) functions as an inhibitor of plant peroxidases. Pep1 protein effectively inhibited the peroxidase driven oxidative burst and thereby suppresses the early immune responses of maize. Moreover, a direct interaction of Pep1 and the maize peroxidase POX12 in vivo was observed, using fluorescence complementation assays, [33].

Catalases occupy a special place in plant–microbial interactions and enhance the virulence of fungal pathogens by reducing the ROS concentration in the zone of infection and suppressing the oxidative burst [2]. Thus, it was shown that the expression of the *CatB* gene encoding fungal catalase was activated in the leaf tissue of the susceptible cultivar at the stage of penetration of the apressoria of the fungus *Blumeria graminis* [34]. Previously, we showed that the aggressive Sn9MN isolate expressed the SNOG_03173.1 gene encoding fungal catalase much more strongly than the avirulent Sn4VD isolate upon inoculation of the susceptible genotype Zhnitsa [35]. Moreover, the transcript level of the SNOG_03173.1 gene increased already after 6 h of infection, and a high mRNA content of this gene was detected 72 h after infection [35]. In the present study, we found the same pattern of changes in expression for the *SnToxA* gene in the Zh/Sn9MN variant. We assume that the increased activity of catalase in susceptible genotypes infected with isolates SnB and Sn9MN is the sum of the activity of fungal and plant catalase and is regulated by NE SnToxA.

Recently chitin binding activity for SnTox1 was visualized in vivo using a GFP tagged version of the protein [8]. It has been shown that SnTox1 binds to the surface of the hyphae particularly near points of hyphal branching or plant penetration. Upon penetration into a plant, various components of the fungal cell walls, such as glucans, chitin and proteins, can be degraded by hydrolytic enzymes of plant origin, such as beta-1,3-glucanases, chitinases, serine and cysteine proteases and then act as a PAMP to trigger major immune responses [1]. For example, the effector protein Ecp6 of *Cladosporium fulvum* mediates virulence through suppression of chitin-triggered immunity in plants. *C. fulvum* Ecp6 (CfEcp6) is secreted at high levels during plant infection and binds chitin, thereby blocking chitin-triggered immunity responses through confiscating chitin fragments [36]. Thus, chitin binding activity of SnTox1 was associated with a prevention of plant chitinases from binding with hyphae and degradation of the fungal cell wall and the release of chitin fragments into the apoplast. Consequently, the development of plant defense reactions was suppressed [8]. In addition, we have shown earlier that chitin fragments activated plant peroxidases, leading to the development of two reactions [37]. In the first place, the rapid activation of peroxidases led to an oxidative burst, in the second place, the slow accumulation of peroxidases around the zone of pathogen penetration in the presence of chitin fragments created conditions for lignification of this zone and restrictions of the pathogen [37]. The results of the present study showed that SnTox1 mainly influenced peroxidase by reducing its activity. Thus, our results suggest that the *Snn1*–SnTox1 interaction reduced the peroxidase activity and the transcript level of gene encoding lignin-forming anionic peroxidase *TaPrx* by binding to chitin.

**Figure 6:**
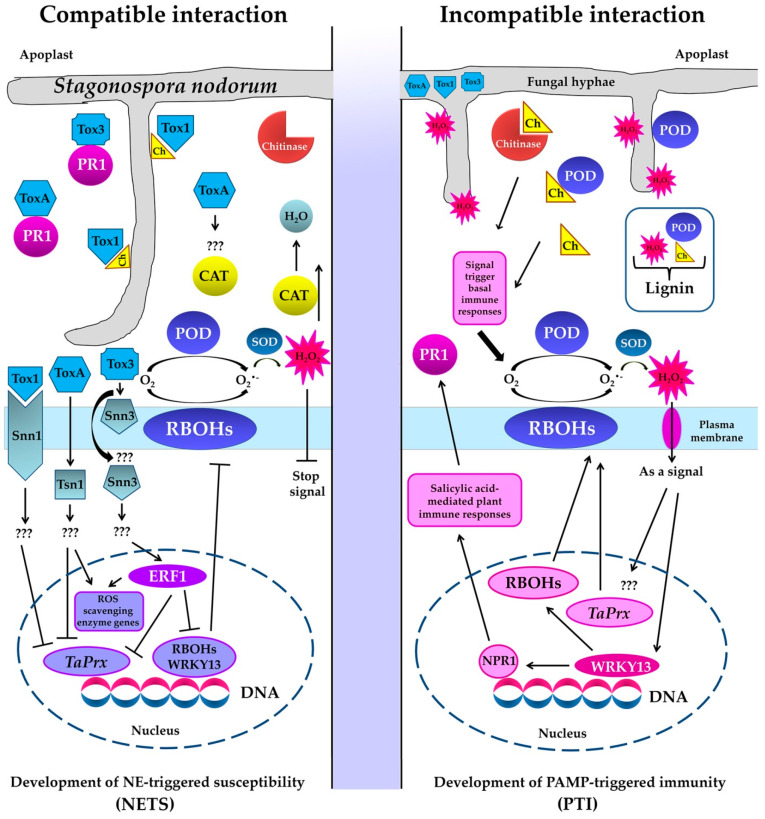
Model illustrating the development of necrotrophic effector-triggered susceptibility (NETS) in a compatible interaction and PAMP-triggered immunity (PTI) in an incompatible interaction with the participation of NEs SnToxA, SnTox1 and SnTox3 and enzymes of redox metabolism of the host plant (NADPH-oxidases localized on the plasmallema also known as respiratory burst oxidase homologs (RBOHs), peroxidases (POD) superoxide dismutase (SOD) and catalase (CAT)) in the *S. nodorum*–*T. aestivum* pathosystem. Legend: Ch—chitin, ERF1—transcription factor Ethylene responsive factor 1, NPR1—Nonexpressor of PR Genes1, *TaPrx*—gene encoding anionic peroxidase, 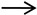
—positive regulation, 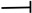
—negative regulation, ???—unknown signal transduction pathway.

A schematic representation of these data illustrating the development of NETS in a compatible interaction and PTI in an incompatible interaction in the *S. nodorum*–*T. aestivum* pathosystem is presented in Figure 6. The model is based on new data generated in this study and previous results of [14] as well as literature data [7,31].

Thus, we have shown earlier that the *Snn3*–SnTox3 compatible interaction triggers the ethylene signaling pathway by inducing the ERF1 transcription factor to suppress the salicylate signaling pathway through inhibition the expression of *WRKY13*, *RBOHs*, and *TaPrx* genes [14]. In addition, Figure 6 shows that in an incompatible interaction, H_2_O_2_ synthesized in the apoplast performs several functions: (1) cytotoxic function—H_2_O_2_ comes into contact with the fungus hyphae, destroys them and restricts their growth; (2) signaling function—H_2_O_2_ penetrates into the cell through special channels, aquaporins, and activates defensive signaling pathways; (3) H_2_O_2_ in the presence of peroxidase and chitin residues creates conditions for lignification of the affected area.

## 4. Materials and Methods

### 4.1. Research Objects

The objects of the study were three cultivars of bread spring wheat (*Triticum aestivum* L.) contrasting in resistance to *S. nodorum* Berk.: Omskaya 35 (Om35), Kazakhstanskaya 10 (Kaz10) and Zhnitsa (Zh). Wheat seeds were obtained from the Bashkir scientific research Institute of Agriculture of Russian Agricultural Academy. Plants were hydroponically grown for seven days on 10% Hoagland–Arnon nutrient medium in a KS-200 SPU growth chamber (Russia) at 20/24 °C (night/day) at the irradiance 146 W/m^2^ FAR (Osram lamps L 36W/77) and the 16 h photoperiod. The pathogenic objects were three isolates of the fungus *S. nodorum*: Sn4VD, SnB and Sn9MN (from the collection of Institute of Biochemistry and Genetics, Ufa Federal Research Centre, Russian Academy of Sciences, Ufa, Russia). All *S. nodorum* isolates were maintained on potato-glucose agar (PGA) at 21 °C and 12 h photoperiod.

### 4.2. Experimental Design

All experiments were carried out on intact 7-day-old seedlings, with the exception of experiments evaluating the resistance of genotypes, which were performed on the separated first leaves [38]. In other cases, 7-day-old seedlings placed in separate vessels were sprayed with suspension of *S. nodorum* isolates Sn4VD, SnB or Sn9MN with 10^6^ spores mL^−1^ in 0.02% Tween 20 to study various parameters. Control plants were sprayed with a solution containing only the wetting agent Tween-20 (0.02%). The volumes of all solutions allowed full moistening of leaves. To study biochemical characteristics, the shoots of intact wheat seedlings were fixed in liquid nitrogen 24 and 72 h after inoculation with *S. nodorum* isolates Sn4VD, SnB and Sn9MN. To study plant-host genes expressions, the shoots of intact wheat seedlings were fixed in liquid nitrogen 6 and 24 h after inoculation with *S. nodorum* isolates Sn4VD, SnB and Sn9MN. In the case of studying the transcriptional activity of *S. nodorum* NE genes, plants were fixed 6, 24 and 72 h after inoculation with *S. nodorum* isolates Sn4VD, SnB and Sn9MN. The variants of treatments and the number of repetitions are indicated in the tables and figures.

### 4.3. Seedling Resistance

Segments of first wheat leaves of 7-day-old plants were placed in Petri dishes on wet cotton wool containing 0.004% benzamidazole (10–12 leaves/dish). Then 5 μL of a spore suspension of *S. nodorum* isolates Sn4VD, SnB or Sn9MN with 10^6^ spores mL^−1^ in 0.02% Tween 20 were spotted onto the leaf surface and Petri dishes with leaves were transferred to the KS-200 SPU growth chamber under controlled conditions. The development of SNB symptoms on wheat leaves was registered on the sixth day after infection with *S. nodorum* isolates using an SP-800UZ Image Stabilization camera (Olympus, Bekasi, Indonesia); the damaged area was measured using the ImageJ 1.44 computer program (rsbweb.nih.gov/ij/download.html, accessed on 15 June 2021) and expressed as a percentage of the total leaf area. In addition, the degree of lesion was also evaluated according to the International scale based on the percentage of the damaged area of plant organs: RR (0–5%)—varieties with very high and high resistance; R (up to 10–15%)—resistant varieties; M (up to 25%)—slightly susceptible varieties; S (up to 40–65%)—susceptible varieties; SS (over 65%)—varieties with very high and high susceptibility. Detriment was also scored on a qualitative scale rating the type of lesion from 0 to 5 as described by [39].

### 4.4. Isolation of DNA and Performing the Polymerase Chain Reaction (PCR)

DNA was isolated from wheat seedlings and 7-day fungus culture by the phenol-detergent method [40]. Identification of NE genes SnToxA, SnTox1 and SnTox3 in *S. nodorum* isolates Sn4VD, SnB and Sn9MN was performed by PCR with gene-specific primers *SnToxA* (JX997419), *SnTox1* (JX997402) [5] and *SnTox3* (FJ823644) [9]. Primers for the housekeeping gene tubulin (S56922) [41] were used as an internal control for the presence of fungal DNA. PCR with the cDNA template was performed in a TP4-PCR-01-Tertsik type PCR machine (DNK-Tekhnologia, Russia).

The dominant allele of the *Tsn1* gene was identified in wheat cultivars by PCR with primers for the *Xfcp623* microsatellite marker on the internal region of the *Tsn1* gene [4]. The dominant allele of the *Snn1* gene was identified with primers for the intragenic marker *Snn1* (KP085710) [5]. The allelic state of the *Snn3-B1* locus was determined by PCR with primers for the *Xcfd20* and *Xgwm234* microsatellite markers [13]. The presence of an amplification product proved the existence of a dominant allele of the gene, the absence of an amplification product indicated the presence of a null (recessive) allele. In all cases, PCR products were resolved in 7% PAAG stained with ethidium bromide using the Gene Ruler DNA Ladder (Fermentas). The gels were photographed using a documenting system of GelDoc XR (Bio-Rad Laboratories, Hercules, CA, USA).

### 4.5. Gene Expression Analysis

Total RNA was isolated from control and infected with *S. nodorum* isolates (Sn4VD, SnB and Sn9MN) first leaves of 7-day-old wheat seedlings (approximately 100 mg from 5 plants) of three varieties Om35, Kaz10, and Zhnitsa, fixed in liquid nitrogen, with TRIzol™ Reagent (Sigma, Germany) according to the manufacturer’s instructions. Analysis of genes expression for NEs (*SnToxA*, *SnTox1* and *SnTox3*) in different isolates of *S. nodorum* during inoculation of wheat plants and expression of genes for wheat oxidoreductases (*TaRbohD, TaRbohF*, *TaSod*, *TaPrx*) was performed by quantitative real-time polymerase chain reaction (qPCR). The potential contaminating DNA was digested with DNaseI (Synthol, Moscow, Russia). First-strand cDNA was synthesized using the M−MLV reverse transcriptase (Fermentas). Oligo (dT)15 was used as a primer, and the reverse transcription reagents were incubated at 37 °C for 1 h in a total volume of 25 µL. After ten-fold dilution, 2 µL of the synthesized cDNA was used for qPCR. Quantitative PCR was performed by polymerase chain reaction in real time using a set of predefined reagents, EvaGreenI (Synthol, Moscow, Russia), and a CFX Connect real-time PCR Detection System device (BioRad Laboratories, Hercules, CA, USA). The qPCR program was as follows: 95 °C for 5 min; 40 cycles of 95 °C for 15 s, 60 °C for 20 s, and 72 °C 30 s. After the final PCR cycle, a melting curve analysis was conducted to determine the specificity of the reaction (at 95 °C for 15 s, 60 °C for 1 min, and 95 °C for 15 s). The efficiency of each primer pair was determined using 10-fold cDNA dilution series in order to reliably determine the fold changes. Real-time PCR was performed using primers for genes encoding two isoforms NADPH oxidase (GenBank Accession No. *TaRbohD*, AK335454), (*TaRbohF*, GenBank Accession No. AY561153) [15], superoxide dismutase (SOD) (*TaSod*, GenBank Accession No. JX398977.1) [42], anionic peroxidase (*TaPrx*, GenBank Accession No. TC151917) [43], *SnToxA* (JX997419), *SnTox1* (JX997402) [5], and *SnTox3* (FJ823644) [9]. To normalize the expression results of the studied genes, primers were used to the tubulin gene of the fungus *S. nodorum* (S56922) [39] and to the gene of constitutively expressed protein that is a wheat ribonuclease inhibitor (RNase L inhibitor-like) *RLI* (GenBank Accession No. AY059462) [44]. The quantification of gene expression was performed using a CFX Connect real-time PCR Detection System (BioRad Laboratories, USA). All reactions, including the non-template control, were performed three times. The threshold values (CT) generated from the CFX Connect real-time PCR Detection System software tool (Applied Biosystems, Foster City, CA, USA) were employed to quantify the relative gene expression using the comparative threshold (delta CT) method. Three independent biological replicates were performed for each experiment.

### 4.6. Biochemical Parameters

To measure the hydrogen peroxide (H_2_O_2_) production and the activity of redox enzymes (POD and CAT), plant material (first leaves of wheat seedlings approximately 150 mg from 5 plants) (1:5 weight/volume) was fixed in liquid nitrogen and then it was homogenized in 0.05 M solution of Na-phosphate buffer (PB), pH 6.2 and incubated at 4 °C for 30 min. Supernatants were separated by centrifugation at 15,000× *g* for 15 min (5415 K Eppendorf, Hamburg, Germany). Concentration of H_2_O_2_ in the supernatant was determined using xylenol orange in the presence of Fe^2+^ at 560 nm by the method of [45]. POD activity was determined by a micromethod in 96-well plates (Corning-Costar, Glendale, AZ, USA) by the oxidation of (o-) phenylenediamine in the presence of H_2_O_2_ at 490 nm on a Benchmark Microplate Reader spectrophotometer (Bio-Rad Laboratories, Hercules, CA, USA) [38]. The enzyme activity was expressed in optical density/mg protein per minute. CAT activity was determined by a micromethod based on the ability of H_2_O_2_ to form a stable colored complex with molybdate salts [38]. Optical density was measured at 405 nm on a Benchmark Microplate Reader spectrophotometer. CAT activity was calculated using a calibration curve and expressed in µM H_2_O_2_/(mg protein per min). Protein content was determined by the Bradford method.

### 4.7. Statistical Analysis

Experiments were performed three times with three replicates for each treatment, except for the measurements of infected areas, which were performed in no less than 30 biological replications for each experiment. One replicate contained shoots of five plants in the case of qPCR and biochemical assay of enzyme activity and H_2_O_2_ production. Experimental data were expressed as means ± SE, which were calculated in all treatments using MS Excel. Analysis of variance (ANOVA) was used to calculate the least significance difference (LSD) at *p* < 0.05 to discriminate means.

## 5. Conclusions

Obtained results indicate that all three NEs of *S. nodorum* SnToxA, SnTox1, SnTox3 played an important role in inhibition of ROS during PTI at the initial stage of infection, despite the fact that at the late stage of infection, all three NEs caused the formation of necrosis and chlorosis on wheat leaves of susceptible genotypes. Our results also showed that inhibition of ROS in PTI by NEs occurred only in the presence of the susceptibility genes *Tsn1*, *Snn1*, *Snn3*. Therefore, our results suggest that effector–host sensitivity gene interactions have the ability not only to hijack the host’s own ETI pathway, but also suppress the host’s own PTI pathway, resulting in NE-triggered susceptibility (NETS). Nevertheless, many more questions remain. What role do genes *Tsn1*, *Snn1* and *Snn3* play in the suppression of PTI? What pathways act after effector is binding to its receptor? What is the main target of the effector in the host? Therefore, the wheat–*S. nodorum* system is much more complex than currently described in the literature, and great additional work is needed to characterize this pathosystem.

## Figures and Tables

**Table 1:** Transcriptional activity of *SnToxA*, *SnTox1*, and *SnTox3* effector genes (*in planta*) in *T. aestivum* cultivars Zhnitsa, Kazakhstanskaya 10, Omskaya 35 with different allelic states of susceptibility genes inoculated with *S. nodorum* isolates SnB, Sn9MN, Sn4VD.

Isolate of *S. nodorum*	Necrotrophic Effectors	Cultivars								
		Omskaya 35 (*tsn1/snn3/Snn1*)			Kazahstanskaya 10 (*tsn1/Snn3/Snn1*)			Zhnitsa (*Tsn1/Snn3/Snn1*)		
		Time after Infection, Hours								
		6	24	72	6	24	72	6	24	72
**Sn4VD** (*toxA/tox3/tox1*)	*SnToxA*	0.05 ± 0.02 ^a^	0.01 ± 0.01 ^a^	0.0 ± 0.0 ^a^	0.07 ± 0.05 ^a^	0.09 ± 0.4 ^a^	0.0 ± 0.0 ^a^	0.04 ± 0.02 ^a^	0.01 ± 0.12 ^a^	0.0 ± 0.0 ^a^
	*SnTox3*	0.01 ± 0.01 ^a^	0.0 ± 0.0 ^a^	0.0 ± 0.0 ^a^	0.0 ± 0.0 ^a^	0.03 ± 0.02 ^a^	0.05 ± 0.04 ^a^	0.0 ± 0.0 ^a^	0.0 ± 0.0 ^a^	0.07 ± 0.05 ^a^
	*SnTox1*	0.0 ± 0.0 ^a^	0.04 ± 0.03 ^a^	0.03 ± 0.01 ^a^	0.04 ± 0.02 ^a^	0.04 ± 0.02 ^a^	0.01 ± 0.01 ^a^	0.04 ± 0.02 ^a^	0.01 ± 0.01 ^a^	0.01 ± 0.02 ^a^
**SnB** (*ToxA/Tox3/tox1*)	*SnToxA*	0.01 ± 0.01 ^a^	0.4 ± 0.1 ^c^	0.17 ± 0.11 ^b^	0.0 ± 0.0 ^a^	0.9 ± 0.1 ^d^	1.2 ± 0.8 ^d^	1.72 ± 0.8 ^d^	1.4 ± 0.9 ^d^	2.8 ± 1.2 ^e^
	*SnTox3*	0.14 ± 0.01 ^b^	0.5 ± 0.1 ^c^	0.16 ± 0.1 ^b^	0.7 ± 0.01 ^c^	1.5 ± 0.3 ^d^	11.0 ± 3.6 ^j^	0.9 ± 0.2 ^d^	1.2 ± 0.4 ^d^	12.1 ± 3.5 ^k^
	*SnTox1*	0.01 ± 0.01 ^a^	0.0 ± 0.0 ^a^	0.0 ± 0.0 ^a^	0.0 ± 0.0 ^a^	0.0 ± 0.0 ^a^	0.0 ± 0.0 ^a^	0.0 ± 0.0 ^a^	0.0 ± 0.0 ^a^	0.0 ± 0.0 ^a^
**Sn9MN** (*ToxA/Tox3/Tox1*)	*SnToxA*	0.01 ± 0.01 ^a^	0.08 ± 0.02 ^a^	0.27 ± 0.1 ^b^	0.0 ± 0.0 ^a^	0.1 ± 0.01 ^b^	1.1 ± 0.5 ^d^	3.5 ± 1.8 ^f^	2.4 ± 0.2 ^e^	5.12 ± 0.8 ^g^
	*SnTox3*	0.03 ± 0.01 ^a^	0.2 ± 0.03 ^b^	0.13 ± 0.04 ^b^	0.0 ± 0.0 ^a^	0.2 ± 0.02 ^b^	0.55 ± 0.1 ^c^	0.5 ± 0.2 ^c^	2.73 ± 0.1 ^e^	17.0 ± 1.6 ^l^
	*SnTox1*	0.01 ± 0.01 ^a^	0.9 ± 0.4 ^d^	0.5 ± 0.1 ^c^	0.5 ± 0.1 ^c^	9.8 ± 2.4 ^i^	2.85 ± 1.5 ^e^	5.6 ± 1.3 ^g^	2.6 ± 0.3 ^e^	7.6 ± 2.2 ^h^

Note: The expression of *SnToxA*, *SnTox1*, and *SnTox3* genes was normalized to the expression of tubulin. Means ± SE (n = 6) marked with similar Latin letters do not differ significantly according to the LSD-test (*p* ≤ 0.05).

**Table 2:** Reaction of *T. aestivum* cultivars Zhnitsa, Kazakhstanskaya 10, Omskaya 35 with different allelic states of the *Tsn1*, *Snn1* and *Snn3* susceptibility genes to inoculation with *S. nodorum* isolates Sn4VD, SnB and Sn9MN carrying a different set of NE genes *SnToxA*, *SnTox3* and *SnTox1*.

Cultivar	Damage Reaction	Isolate of *S. nodorum*		
		Sn4VD (*toxa/tox3/tox1*)	SnB (*ToxA/Tox3/tox1*)	Sn9MN (*ToxA/Tox3/Tox1*)
Omskaya 35 (*tsn1/snn3/Snn1*)	Necrosis, %	0.05 ± 0.002 ^a^	5 ± 0.7 ^bc^	23 ± 2 ^e^
	Chlorosis, %	0 ± 0 ^a^	3 ± 0.5 ^b^	0 ± 0 ^a^
	Damage zone, %	0.05 ± 0.001 ^a^	8 ± 1 ^c^	23 ± 2 ^e^
	Damage score	1	2	3
	Resistance group *	RR	R	M
Kazahstanskaya 10 (*tsn1/Snn3/Snn1*)	Necrosis, %	0.05 ± 0.002 ^a^	16 ± 2 ^d^	31 ± 3 ^f^
	Chlorosis, %	0 ± 0 ^a^	35 ± 3 ^f^	25 ± 2 ^e^
	Damage zone, %	0.05 ± 0.001 ^a^	51 ± 5 ^g^	56 ± 4 ^g^
	Damage score	1	4	4
	Resistance group *	RR	S	S
Zhnitsa (*Tsn1/Snn3/Snn1*)	Necrosis, %	1 ± 0.1 ^b^	18 ± 2 ^d^	27 ± 2 ^e^
	Chlorosis, %	2 ± 0.2 ^b^	55 ± 4 ^g^	57 ± 5 ^g^
	Damage zone, %	3 ± 0.3 ^b^	73 ± 6 ^h^	84 ± 6 ^i^
	Damage score	1	5	5
	Resistance group *	RR	SS	SS

Note: * RR (0–5%)—varieties with very high and high resistance; R (up to 10–15%)—resistant varieties; M (up to 25%)—slightly susceptible varieties; S (up to 40–65%)—susceptible varieties; SS (up to 90–100%)—varieties with very high and high susceptibility. Experiments were carried out on the separated first leaves. Means ± SE (n = 30) marked with similar Latin letters do not differ significantly according to the LSD-test (*p* ≤ 0.05).

## Data Availability

Data available in a publicly accessible repository.
